# Compliance with the Framework Convention on Tobacco Control in Slovakia and in Finland: Two Different Worlds

**DOI:** 10.3390/ijerph17186661

**Published:** 2020-09-13

**Authors:** Barbara Pavlikova, Lenka Freel, Jitse P. van Dijk

**Affiliations:** 1 Department of Labor Law and Social Security Law, Faculty of Law, Comenius University, 810 00 Bratislava, Slovakia; lenka.freel@flaw.uniba.sk; 2 Department of Community and Occupational Medicine, University Medical Center Groningen, University of Groningen, 9713 GZ Groningen, The Netherlands; j.p.van.dijk@umcg.nl; 3 Graduate School Kosice Institute for Society and Health, Faculty of Medicine, P.J. Safarik University in Kosice, 040 01 Kosice, Slovakia; 4 Olomouc University Social Health Institute, Theological Faculty, Palacky University, 771 47 Olomouc, Czech Republic

**Keywords:** Slovakia, Finland, tobacco, non-compliance, FCTC

## Abstract

The Framework Convention on Tobacco Control (FCTC) developed by the State Parties to the World Health Organization was ratified in Slovakia in 2004 and in Finland in 2005. The aim of this study was to explore and compare compliance with the FCTC in Finland and Slovakia. This is a two-country comparative study of tobacco control policy based on implementation of the FCTC in Slovakia and Finland. Compliance with the FCTC was measured similarly in Slovakia and Finland in terms of their institutional structure supporting a smoking free environment and implementation of selected articles of the FCTC. In Finland the responsibilities for anti-tobacco policy are clearly assigned. Slovakia does not have specifically responsible institutions. Finland has a clear plan for achieving the goal of a smoking-free country based on empirical evidence. Slovakia meets only the minimum standard resulting from its commitment as ratified in the FCTC and data are out of date or missing completely.

## 1. Introduction

The Framework Convention on Tobacco Control (FCTC) is the first global public health treaty negotiated under the auspices of the World Health Organization (WHO). It provides an internationally coordinated response to combat the tobacco epidemic [1]. The FCTC was adopted in 2003 and came into force in 2005 [2]. As of August 2020, the FCTC has 168 signatories and 182 Parties. The European Union itself also adopted the Convention on 30 June 2005. FCTC was open for signature until 29 June 2004. Countries wishing to become a Party, but which did not sign the Convention by that date, may still do so by means of accession [3]. Parties are committed to implementation of the FCTC and its five main areas of interest as identified by the creators of the Convention: effective tax and price policy; restriction of advertising and promoting of tobacco products; smoking bans; package warnings; combating the illicit trade in tobacco [1,4].

The Conference of the Parties (COP) is the governing body of the FCTC and comprises all Parties to the Convention [5]. The eighth session of COP to the FCTC (COP8) took place in October 2018 in Geneva, Switzerland [6,7]. This session approved a Medium-Term Strategic Framework (MTSF) known as the Global Strategy to Accelerate Tobacco Control. It is intended to strengthen implementation of the FCTC and to guide the work of all involved entities concerning tobacco control from 2019 to 2025 [8,9,10,11]. A Party-driven pilot project for an Implementation Review Mechanism (IRM) was due to start in 2019, helping Parties understand where they might improve their tobacco control policy [12].

Tobacco control has become one of the key pillars of the Global Non-Communicable Diseases Strategy, emphasizing prevention through influencing the four main behavioral risk factors: tobacco use, unhealthy diet, physical inactivity, and harmful use of alcohol [13,14]. With respect to the impact of tobacco on development, proper implementation of the FCTC is considered to be one of the key factors in achieving the Sustainable Development Goals (SDGs) [15]. The FCTC 2030 project, funded by the United Kingdom and Australia, is another tool assisting the Parties in this implementation. The Parties can receive official development assistance (ODA) to achieve the SDGs by advancing implementation of the Convention. The ODA project runs from April 2017 until March 2021 [16,17].

Slovakia signed the FCTC on 19 December 2003 and ratified it on 4 May 2004. In Finland, the FCTC was signed on 16 June 2003 and it was ratified on 24 January 2005 [18]. The Additional Protocol to Eliminate Illicit Trade in Tobacco Products is in progress in both countries: Slovakia acceded to the Protocol on 25 September 2017, and Finland signed it on 23 September 2013 but has not approved it yet [18].

According to the 2018 Global Progress Report on Implementation of the FCTC, progress towards implementation of the different articles varies between 13% and 88%. Some articles are implemented more successfully (articles 8, 11, 16), while there is little or no progress in implementation of others (articles 17, 18) [19]. The needs of people from segregated communities still need to be addressed in order to reduce the high prevalence of tobacco use in such communities [20,21].

Slovakia submitted its latest report on the current status of implementation of the FCTC in December 2018. The 2018 report showed there were 36% current smokers in the overall population, consisting of 26% daily smokers and 7% occasional smokers (males and females) during the reporting period (2016–2018), which the report highlights as a pattern of declining consumption. Among young smokers, 11% were current smokers. However, many additional data are lacking and the information dates from 2013–2014. The authors did not identify any gap between needs and resources [21]. Slovakian tobacco control policies are considered by the OECD to be less comprehensive than in many other European Union (EU) countries [22].

Finland submitted a report in March 2018 after having done so previously in 2016. The data cover the two-year period from 2016 to 2017. The country indicated 23.1% current smokers, consisting of 13.0% daily smokers and 10.1% occasional smokers. Tendencies differed in each group: in general, the prevalence of daily smokers and never smokers decreased, while the prevalence of occasional smokers and of former smokers increased. There were 3.7% young current smokers aged 14 years among boys and girls and 13.9% of 16-year-olds. No gaps between needs and resources were identified. The report provides relatively comprehensive information on the recent status of the WHO FCTC implementation in Finland [23].

It is important to understand what “non-compliance” means. Concepts of “compliance” and “non-compliance” in international law and health care seem to be problematic and difficult to explain [24,25,26,27,28]. In general, compliance means the ability or willingness to act according to an order, set of rules, or request [29]. In a more objective meaning compliance is understood as the implementation and enforcement of all aspects of the treaty; however, non-compliance can have a continuing nature, whereby actual implementation of provisions in the national context can be merged with the intent or actions taken to implement (steps towards full implementation). Kingsbury [30] emphasizes that the meaning of compliance strongly depends on the theoretical resource, so different theories lead to significantly different notions about the concept. Guzman [31] states that international law can affect state behavior because of the threat of sanctions following its violation. Von Stein [32] understands compliance as the degree to which state behavior conforms to what the agreement prescribes or proscribes. Non-compliance is then defined as conflict with such prescription or proscription (expected behavior). We use this definition in this study.

While speaking about compliance with international rules, we will also discuss the influence of enforcement. Enforcement can be understood in the sense that a regulator must enforce compliance with rules. Enforcement is as much about investigating, gathering, and sharing information as it is about imposing penalties. It consists of the following phases: inspection (realized by e.g., the Conference of the Parties of the FCTC at the national level); investigation powers (e.g., the National Supervisory Authority for Welfare and Health in Finland); surveillance powers (e.g., realized through bi-annual reports submitted by the FCTC Parties or by the Regional Health Authorities in Slovakia); the imposition of corrective or remedial action (e.g., the National Supervisory Authority for Welfare and Health in Finland or the Labor Inspectorate in Slovakia); the imposition of penalties (e.g., the Slovak Trade Inspection or the State Veterinary and Food Administration) [29]. In a different sense it involves the existence of sanctions or some material consequence if non-compliance occurs [32].

When national and international rules are conflicting, a principle of pacta sunt servanda (Article 26 of the Vienna Convention on the Law of Treaties) is applied, according to which a state is under the duty to honor its international obligations even if it means changing its national law. Once the state has ratified a treaty, it cannot successfully amend its domestic legislation with a view to avoiding compliance with international obligations. In such a situation, international law prevails over national law. As stated in the United Nations Headquarters opinion, on the international legal plane, national law cannot derogate from international law. The typical way of harmonizing national and international rules is to transform (incorporate) an international treaty into national laws, so that it is applicable and enforceable in the national state [33]. National institutions are then responsible for implementation and enforcement of the rules governed by the international treaty incorporated into national laws.

Evidence about the implementation of the FCTC in Slovakia is very scarce, while much more information is available for Finland. The aim of this study was to explore, assess, and compare compliance with the FCTC in Finland and Slovakia.

## 2. Materials and Methods

### 2.1. Sample

Our design is a case study, with the current status of implementation of the FCTC in Slovakia and in Finland as the subject. Slovakia and Finland have several similar characteristics, which allow us to compare their tobacco control policy. They have a very similar number of inhabitants. Both countries are Members of the EU, the Eurozone, Schengen Area, the Council of Europe, and the OECD. Their state establishment is a parliamentary republic, and both have a comparable position on the Human Development Index. Documents were obtained from the official websites of state authorities, available statistics, and laws. For our documentary study no Ethics Committee approval was necessary.

We worked with the official websites of the Public Health Authority of Slovakia, Regional Public Health Authorities, Slovak Regional Office, League against Cancer, and the Slovak Medical Society. For Finland, we used mainly the websites of the National Institute for Health and Welfare, Valvira, the Ministry for Social Affairs and Health, and Action on Smoking and Health (ASH Finland), as well as the Tobacco-free Finland 2030 network, Filha, Finnish Federation for Social Affairs and Health (SOSTE), Savon Sydänpiiri, and Duodecim. We also used information published on official sites of other state authorities and non-governmental bodies.

In addition to the text of the Framework Convention on Tobacco Control and fundamental non-legislative documents, action plans, and declarations, we worked with the most important laws in both compared countries: Act no. 377/2004 Coll. on the protection of non-smokers as amended (Slovakia) and Tobacco Act no. 549/2016 as later amended (Finland).

Reports submitted bi-annually by the national governments to report on FCTC implementation present the main source of data relating to smoking rates in both countries. Finland submitted the Core Questionnaire on 15 March 2018 (78 pages) under the auspices of the Ministry of Social Affairs and Health. Slovakia submitted the last Core Questionnaire on 4 December 2018 (72 pages) under the auspices of the Public Health Authority. The biggest difference between these two reports is in the content: Finland in its report provides much more accurate data on smoking than Slovakia, which left most of the fields empty.

All three authors—B.P., J.P.v.D., and L.F.—worked together on selection of the most important information. B.P. together with J.P.v.D. made an overview of available sources and data. In the next step, B.P., J.P.v.D., and L.F. agreed on specific articles of the FCTC which were analyzed. B.P., as the first author prepared the comparison and the analysis, and J.P.v.D. and L.F. added their points of view, checked the accuracy of the data, and filled in the missing parts. The distribution of role is described at the end of the article in more detail.

### 2.2. Measures

Compliance with the FCTC was measured similarly in Slovakia and Finland in terms of their institutional structure supporting a smoking free environment and implementation of selected articles of the FCTC.

We measured the institutional framework by counting the number of functional governmental and non-governmental institutions in Slovakia and in Finland whose task it is to implement, develop, or control their anti-tobacco policy. We analyzed the public and private sectors to the extent they are included in the WHO FCTC implementation.

We measured compliance with the FCTC in terms of the levels of enforcement and interest in respecting its international rules. We focused on inspection and investigation powers, surveillance powers, power to impose corrective or remedial actions, and power to impose penalties in the field of tobacco control policy. Non-compliance was also evaluated on the basis of the implementation level of selected articles of the FCTC. We examined these articles: 5 (5.1—documents, 5.2a—coordination mechanism, 5.4 and 5.5—cooperation, 5.6—funding); 8 (protection against exposure to smoke); 12 (education and communication); 14.2 (treatment); 16 (sale of tobacco products to minors); 19 (responsibility); 20, 21, and 22 (research, exchange of information, and data availability) and compared the compliance with them in Slovakia and in Finland. This selection was made because we considered these articles as being influential with regard to tobacco control, as well as being comparable for both countries.

### 2.3. Reporting

We focused firstly on the structure of institutions governing tobacco control policy in both countries. Governmental and non-governmental bodies with some competences in tobacco control policy are covered. Then we paid attention to the compliance with domestic and international commitments in the field of tobacco control policy, and to the enforcement of rules already adopted in both countries.

## 3. Results

### 3.1. Institutional Framework

In Slovakia the number of functional state authorities and executive bodies (governmental and non-governmental) in the field of anti-tobacco policy is one of the questionable issues. Shortly before and after the adoption of the FCTC in 2004 a simple institutional structure was established, which consisted of governmental and non-governmental authorities. In the meantime, most of them have been dissolved (Table 1).

In Finland the tobacco control policy has been developed since 1976 and is supported by numerous active institutions with functional and regularly updated websites (Table 1).

### 3.2. Non-Compliance from the Point of View of Enforcement and Implementation

#### 3.2.1. Inspection, Investigation, and Surveillance Powers

In Slovakia the main controlling authorities are the Ministry for Health, Labor Inspectorates in cooperation with Regional Health Authorities, the Ministry for Defense, the State Veterinary and Food Administration, and the Slovak Trade Inspection, but other institutions such as municipalities, the Police, and regular transport operators are also included (Table 2). In 2012, 43% of participants in a survey with 500 respondents older than 18 years [34] declared that they had witnessed a violation of the ban on smoking at public transport stops and 85% of interviewees considered the checks to be insufficient.

In Finland the monitoring and supervision is governed by Valvira (the National Supervisory Authority for Welfare and Health), which assists in achieving the goals stated in strategies at the requested level. All sellers of tobacco products and nicotine-containing liquids are subject to a license (retail sale) or to a notification (wholesale) from the relevant local authority. Sellers are also charged an annual supervisory fee per selling point according to an annually agreed tariff [35].

#### 3.2.2. Imposition of Corrective, Remedial Actions, and of Penalties

The Slovakian Act on Protection of Non-Smokers differentiates between administrative misdemeanors and offenses. Depending on the severity and nature of the breach of the law, the responsible institutions may impose a fine on the entity from €331 to €331,939. The total amount of fines for the period 2012–2014 imposed in administrative procedures was €630,999 [34].

In Finland Valvira is responsible for the supervision to ensure that the above-mentioned products are legally compliant. If they are not, the authority may issue a notice of a conditional fine and require that the illegal product be withdrawn from the market [36]. Valvira has not issued any fines concerning any breaches of the implementation of the FCTC [37].

### 3.3. Implementation of Selected FCTC Articles into Practice

The situation regarding the implementation of articles 5.1 (development, implementation, periodic updating and reviewing of comprehensive multi-sectoral national tobacco control strategies, plans, and programs) and 5.2 letter a (establishment or reinforcement and financing a national coordinating mechanism or focal points for tobacco control) differs in Slovakia and Finland. A well-functioning institutional structure with an array of strategic documents and action plans exists in Finland, whereas Slovakia has not adopted documents enabling a clear institutional framework to be created or any concrete instructions to be followed step by step, as can be seen in Table 1.

Slovakia is less active than Finland in the field of international, intergovernmental, and regional cooperation and exchange of information (articles 5.4, 5.5, 20, 21, and 22). Comparing the answers to the 2018 Core questionnaire of the reporting instrument of FCTC [21,23], submitted by both countries, especially in its part “D” (International cooperation and assistance), the Slovakian answers were completely negative (the country was not cooperating internationally and did not need assistance), while the Finnish were almost all positive. Overall, the informative value of the answers provided in the questionnaire is incomparable, as the Slovak representatives left many boxes empty or used the phrase “not applicable”.

Financing the implementation process and related commitments is always an issue. In accordance with article 5.6 of the FCTC, Parties are obliged to find appropriate financial resources. In its report from 2016 [34] Slovakia indicated that no resources were allocated from the budget of the Ministry for Health of the Slovak Republic for the preparation and publishing of educational materials (art. 12 of the FCTC) or for material and technical equipment of smoking cessation counseling centers (art. 14.2 of the FCTC).

Finnish non-governmental organizations in particular which are core in the implementation of anti-tobacco policy also face a lack of designated funds for tobacco control, which increases their dependency and they often have to seek funding on an annual basis [38].

Education, practice, and communication (art. 12 of the FCTC) seems to be a weak part of FCTC implementation in general in Slovakia. In 2012 only 9% of survey participants had knowledge of the Act on Protection of Non-Smokers, and a large number of them requested better exchange of information by means of TV (69%), printed media (63%), or radio (55%) [34]. Almost all realized that the measures and campaigns were aimed at children and youth.

In Finland mass-media campaigns are evaluated as ineffective, mainly if they are non-targeted and not considered to be a priority of national institutions [38]. Warning media campaigns targeting the whole population are not part of a Comprehensive Tobacco Control Program, but there exists utilized media planning, and assessment of implementation and effectiveness [39].

The implementation of article 16 of the FCTC (ban on the sale of tobacco products to minors) may be used as another example. In 2013 and 2014 control operations with dummy customers were carried out in Slovakia by the Slovak Trade Inspection. In 2013 and 2014 a lot of tobacco products were still sold to minors. The decrease since 2014 could be explained by the changing behavior of minors [34,40].

In Finland, buying cigarettes on behalf of a minor became an offense punishable by up to six months in prison in 2016. Even selling one cigarette to a minor is interpreted as a tobacco-selling violation, and it is also forbidden to offer tobacco without payment to minors, but this is not punishable [41]. There has been a permanent decrease in tobacco purchasing from commercial sources since 1995: the purchase rate among 14-year old smokers diminished from 90% to 67% in 2003. Purchases in shops decreased most among 14-year-olds: from 39% to 14%; purchases in kiosks decreased from 70% to approximately 35% [42]. The overview of the practical implementation of the selected articles in both compared countries is presented in Table 3.

### 3.4. Slovak and Finnish Compliance with International and Domestic Rules in General

Slovakia is a defendant party before European judicial institutions from time to time. In 2017 it lost 12 cases at the European Court of Human Rights (ECHR) in Strasbourg. The complainants are entitled to compensation of €5.8 million in total, because Slovakia has not fulfilled its obligations from 2014, and other breaches of legal duties were also discussed [43,44]. Slovakia’s general compliance with law may also be clear from its many appearances as a defendant party before European judicial institutions. In 2017, it not only lost the above-mentioned 12 cases, but also one at the European Court of Justice (ECJ), and it was sued by the European Commission 12 times between 2007 and 2016. During that period of time it lost four cases [45,46,47]. All Slovak general courts and the Constitutional Court failed to respect the case law of the ECHR [48]. The most important cases which Slovakia has lost at the ECHR were cases of violation of Roma women’s rights in involuntary sterilization cases [49,50]. The ECJ has ruled against Slovakia in some high-profile cases on illegal landfills [51,52,53], non-payment of Christmas pensions to citizens living abroad [54], and for the impossibility of appealing to a court against a decision to refuse, annul, or revoke a visa in the case of visa applicants [55,56]. The EC has opened a total of 48 cases on transposition of EU directives by Slovakia [57,58].

Finland is sometimes also a defendant party in European judicial proceedings. Between 2016 and 2018 there were three requests granted before ECHR [59]. In 2016 it had no repetitive or leading cases, but in 2017 there were two [60]. More than half of the findings of violations from previous years concerned Article 6 of the Convention (right to a fair trial), essentially with regard to length of proceedings [61].

The main barriers to even better compliance with the rules supporting a smoking-free environment in Finland are considered to be insufficient funding, differing views of political representatives, disparities in tobacco use, and deficiencies in smoking cessation services [38].

The main barriers to compliance with the law in the field of tobacco control in Slovakia are the lack of supportive documents, unclear institutional structure, and control mechanisms aimed mainly at sales control of tobacco products to minors. Starting from the Core Questionnaire, the reporting instrument of FCTC [21], it seems that anti-tobacco policy is not a priority in Slovakia, in contrast to Finland where all accessible sources are focused on building a smoking-free country. In the case of Slovakia, the lack of capacity to carry out international duties can be seen as the reason for the current status of the FCTC implementation [62]. Compliance with expected behavior can be a matter of motivation, self-interest, and reputation.

## 4. Discussion

The FCTC has entered the second decade of its operation, so lessons learned from the first decade can be summarized regarding the role of the institutions, funding, and persisting barriers. Craig et al. [63] stated that the FCTC catalyzed the creation of a national tobacco control law. They also stated that the FCTC strengthened existing tobacco control policies; mobilized and strengthened collaboration between health and non-health sectors and engagement with civil society organizations; mobilized a global tobacco control movement through international cooperation and information exchange. Finally, they stated that the FCTC had prompted governments to take measures to protect tobacco control against vested interests of the tobacco industry, and provided a supporting evidence-based legal framework to overcome challenges to tobacco control measures by the tobacco industry and others. On the other hand, slow progress in reducing the affordability of tobacco products, a lack of funding for enforcement activities and cessation services, illicit trade, and lack of effective measures to support tobacco farmers in switching to alternative livelihoods represent persisting barriers. Additionally, the slow integration of the FCTC into national law, suboptimal enforcement of existing laws, intensified marketing by the tobacco industry, industry interference with policy-making, inadequate resource allocation for tobacco control, and insufficient human capacity to lead implementation efforts remain challenges [64]. However, with the FCTC being a binding instrument, usually ratified by the Parliament, tobacco control has become not only a health issue and a function of the ministry of health, but also an administrative and legal responsibility of different sectors of government [65].

The significant role of stakeholders, especially legislative bodies and non-governmental organizations (NGOs), has been proven, but every authority that is in contact with large transnational tobacco companies is important, because of their strong influence on the national tobacco policy [66]. As a result of a report in which the industry marked WHO as “the leading enemy”, WHO declared that the industry could not be seen as a credible stakeholder in the FCTC process and banned them from participating in the negotiations, even as observers. This requires surveillance and strong advocacy on the part of both governmental and non-governmental stakeholders. NGOs were essential in supporting FCTC negotiations and working to ensure strong implementation of the treaty. NGOs have been particularly good at “shaming and blaming” countries that fail to live up to public health standards. In addition, evidence-based control measures are needed [67].

Funding, together with legislation, has also been a critical issue during the first decade. Countries must develop national capabilities to fund their national tobacco control programs over the long term. Earmarked tobacco taxes provide one effective approach to raising money for tobacco control, while at the same time promoting tobacco cessation [67]. Additionally, as Paaso, Director of the Department for Wellbeing and Services at the Finnish Ministry of Social Affairs and Health, declared: “One of our most important lessons learned in tobacco control is the fact that legislation is the foundation for everything” [68].

The Global Strategy to Accelerate Tobacco Control was developed following a decision of the Parties at the Seventh Session of the Conference of the Parties, which is intended to guide the implementation of the WHO FCTC for the years 2019–2025, including the activities/work of the Parties, the Convention Secretariat and other stakeholders, and to serve as the basis for work planning and budgeting for the 2020–2021, 2022–2023, and the 2024–2025 biennia. It consists of several strategic goals and strategic, operational, and specific objectives [64]. In addition, the Global Strategy contains 20 indicators which refer to progress in the implementation of the WHO FCTC, and the support and assistance provided to the Parties and to the Convention [69].

A controversial dispute has been solved concerning several Australian measures that require the use of plain packaging for all tobacco products in the context of World Trade Organization (WTO) law and implementation of the FCTC [70]. The Agreement on Technical Barriers to Trade (TBT Agreement) is probably the most important piece of WTO law when it comes to assessing national tobacco control measures. The question is whether the FCTC and its guidelines can in fact be regarded as relevant international standards under the TBT Agreement. The WTO adjudicating bodies have not yet analyzed whether the FCTC could be considered a “relevant international standard” within the framework of the TBT Agreement, so there is no case law which has determined whether the FCTC and its guidelines are currently relevant international standards [71].

We explored the compliance of Slovakia with its own laws focusing on tobacco control policy and looked at the reasons for the more successful implementation of the FCTC in Finland. In comparison with Finland, Slovakia as a state tends to be unconcerned with rules in the field of tobacco control. If there is a threat of financial penalty, the state is willing to make some cosmetic changes to meet the requirements related to the imposition of a fine. The weaker sanctions typical for the international norms and the question of reputation have no evincible influence on the behavior of the Slovak authorities. The results from Finland show that a systematic approach and effective institutional structure contribute to successful implementation of anti-tobacco policy.

### 4.1. Institutional Structure

We found no identifiable institution in Slovakia responsible for monitoring implementation of anti-tobacco policy, and the system of control is also fragmented. This is different from Finland, where powers are clearly divided between the governmental and non-governmental sector. Responsibility is demonstrable and competences focus specifically on a smoking-free environment in Finland. Existing institutions in Slovakia, regardless of the sector, deal with drug abuse in general, and tobacco policy is only one and a very small part of the national drug policy.

We agree with Mugwagwa et al. [72] that there is a need for policy implementation rather than developing other additional policies when the first step does not work properly. The position of government, strengthened by strong mechanisms and including appropriate funding, could reinforce the efficient implementation of adopted policies. This opinion is also supported by Esa [73], who considers a transparent institutional framework and consistency and continuity in policy as the key factors for successful policy implementation.

An effective institutional framework plays a significant role in ensuring that a treaty is accepted and is implemented once it has been transformed into national law. Therefore, there is a need for a multi-sectoral interdisciplinary institution (secretariat or committee) to be established. It would be of great importance in building the critical mass necessary to change the priorities of governments. Furthermore, such an institution would be able to add input into the treaty making process by providing insights into the country situations. If it is not possible to create specific institutions for the purposes of the tobacco control process, already existing institutions could be utilized to advance the cause [33].

### 4.2. Compliance

We examined compliance from the point of view of inspection, investigation, and surveillance powers. We found that a complex structure of controlling bodies exists in Slovakia, but it seems that inspections are not carried out very often (or data are still not available) and the enforcement of existing rules is weak. The extent of state control is very narrow, primarily checking compliance with the ban on sale of tobacco products to minors. Findings in relation to inspections and control are contrary to article 8 (protection against exposure to tobacco smoke) and 19 (responsibility) of the FCTC [33]. The data used in the new report for WHO from December 2018 are still from the 2013–2014 period, and judging from some answers it appears the questions were misunderstood.

In Finland, the key institution of surveillance in tobacco control policy is Valvira, whose structure is relatively simple and effective. Furthermore, the general population’s obedience with regard to state authorities and given rules is much higher in Finland than in Slovakia, although there are also differences between particular groups of people concerning whether compliance with the law is stronger in groups than in the individual conscience [74].

Regarding compliance, Franck [75] stated: “Almost all nations observe almost all principles of international law and almost all of their obligations almost all of the time”. The degree of compliance differs from state to state and depends on various key factors. The correlation between the strength of the state and the level of compliance has not been demonstrated. Different variables can also influence non-compliance with international and national norms, such as ambiguity of rules, asymmetry between states, or consent given by different states. Their interpretation of major theories such as realism, liberalism, and constructivism is also crucial [76]. Slovakia tends towards realism (self-interest) more than towards liberalism (trust in democratic values and institutional support), with a few aspects of constructivism (calculation of interest in what is considered as acceptable behavior). Finland can be seen as an example of liberalism. Kosař and Petrov [77] stated that the level of compliance depends on a repeated balancing exercise, in which domestic political actors balance the domestic political costs of compliance against the international reputational costs of non-compliance. Cerna [78] summarizes different approaches influencing policy implementation. She points out that there are around 300 potential variables which can affect the success of policy, and that no ‘one-size–fits-all’ solutions exist. Countries’ compliance varies across issue areas, regions, and time [79]. Fariss [80] demonstrated that there is a positive relationship between ratification of human rights treaties and higher levels of respect for human rights, and thus also of compliance.

### 4.3. Implementation of Selected Articles in Practice

We found that Slovakia has more or less implemented selected articles into its legislation, however the step from legislation into practice has been paid less attention. Looking at the data provided in the circle report, Slovakia appears not to be so active in the field of common international support and cooperation with the aim of achieving the goals within tobacco control policy. Slovakia has indicated that it does not identify any gap in technical or financial resources [21], although these are the fields in which more implementation failure can be identified. In line with this, we found that almost no finances were allocated by the state to the area of education, practice, and communication, nor to the area of material and technical equipment of counseling centers for smoking cessation. The WHO stated in its Global Progress Report from 2017 [19] that 60% of the Parties identified specific needs and gaps in the implementation process. Slovakian reality confirms the existence of gaps, especially in funding, as our findings show, but they remain officially undeclared. However, the support of other Parties and WHO itself can be a key factor in the process of more detailed implementation of the FCTC in Slovakia.

Finland is more successful in implementing the compared articles, and its identification of small or no gaps [23] is therefore more realistic. The number of fines cannot be compared, since Valvira in Finland has declared no imposed fines for violations of the FCTC. The number based on violations of transposed FCTC rules in Slovakia is relatively high. This illustrates the approach of institutions and the commercial or private sector to complying with the given rules. The Finnish Ministry for Social Affairs and Health also appointed a working group in 2017 whose given task was to promote the objective of ending the use of tobacco and other nicotine-containing products by 2030 [81]. Despite its achievements to date, Finland also struggles with lack of financial support, differences in political backing for a smoking-free environment, and need for implementation of structural changes, as Timberlake et al. pointed out [38].

### 4.4. Slovak and Finnish Compliance with International and Domestic Rules in General

Finally, we found that the implementation of the FCTC in Slovakia is one of the examples in which the rules appear better on paper than in practice. Some successes have been achieved, especially in the area of package labeling and tax control policy. The situation in Slovakia is similar to other countries [82,83]. The measures of compliance control in tobacco policy are based on restrictions and bans, not preventive actions and raising public awareness of the side-effects of tobacco use. Smoking is still widespread and generally tolerated, and there is no evidence of reporting violations of the legislation by the public, for instance in cases of smoking at bus stops or on balconies [84].

Finland is a Party to judicial proceedings at the European level, as our findings show. The number of presented cases is lower than for Slovakia, but their content is similar and the countries tend to breach the same articles of international conventions or have problems with transposition of European secondary law. The numbers show greater willingness of the country to fulfil its obligations in the case of Finland.

Compliance is a complicated issue in general. As Bayram [85] shows, existing approaches neglect the heterogeneity inherent in decision-makers’ subjective beliefs in the legitimacy of international law. This is critical for explaining who exhibits a sense of obligation and has a non-egoistic preference for compliance. He used the concept of cosmopolitanism: politicians with a high degree of cosmopolitanism are driven by a sense of legal obligation which results in a social preference for compliance, while those low on cosmopolitanism lack the same sense of normative respect.

Lessons learned show that in the public health field, stronger alliances of advocates and health professionals and better tools and capacity to monitor and report current marketing practices and trends can be very helpful in achieving the goals [86]. Moreover, a similar approach as in the case of WTO rules can be applied to WHO conventions: compliance-related issues must be viewed in a broader perspective, which transcends narrow legalistic views and accounts for the multifaceted interests of WTO members, and the differences between them [87]. As Bachrach and Baratz [88] warn, there are four barriers to be identified in any policy-making process: community values, procedures and institutions, the decision-making process itself, and the implementation process. When the interests of a coalition of different groups meet at a certain moment, it is possible to overcome all these barriers. Policy is also more likely to succeed if the issue originates within government than coming from outside [89].

Regarding the imposition of sanctions, not internally but on an international level, as a “punishment” for undesirable conduct, Gravely [90] argues that it is difficult to achieve effective multilateral sanctions, and those on a bilateral level are more effective. Non-compliance can carry sanctions or some material consequence in the absence of a centralized enforcement authority [32].

### 4.5. Socioeconomic Situation and Poverty as an Influential Factor

Socioeconomic situation (SES) is associated with the prevalence of smoking. As Haustein [91] stated, tobacco and alcohol use are more prevalent in lower socio-economic groups of society. Tobacco and low SES create a vicious circle. Poor families spend a larger proportion of their income on tobacco. Tobacco users are at much higher risk of falling ill and dying prematurely of cancers, heart attacks, respiratory diseases, and of additional costs for health care [92,93]. SES of Slovakia and Finland differs.

According to the OECD [94], the Slovak economy was rising in 2019. However, structural unemployment remains high, and spending by government on active labor market policies is low. Labor market participation of women of child-bearing age is also low, impairing their career and earning prospects.

In Finland, accelerating output growth and measures to curb government spending together are stabilizing public debt. Finland’s employment rate is markedly lower than in the other Nordic countries. The combination of different working-age benefits, childcare costs, personal income taxation, and social security contributions creates complexity, reduces work incentives, and holds back employment [95].

### 4.6. Strengths and Limitations

We explored the barriers to implementation of the FCTC in Slovakia, using the comparison with the implementation of anti-tobacco policy in Finland. This case study provides deeper insight into the structural background of tobacco control policy in Slovakia and points out the biggest challenges in the ongoing process of the efforts to reduce tobacco consumption. Finland was used as an example of good practice as it plans to achieve a smoking-free environment in 2030. We condensed information from various available sources to present an overview of selected aspects of Slovakian tobacco control policy, which are often difficult to find. Information on FCTC implementation in Slovakia is very fragmented, and apart from in WHO reports it is not available in English.

As a limitation we consider the problematic availability of relevant sources and outdated information, and the fact that some of our questions focusing on smoking were unfortunately answered with completely missing data. The contact person responsible for developing tobacco control policy at the governmental level should have completed those data.

### 4.7. Implications

We found that there are differences present in the process of implementation of the FCTC in Finland and in Slovakia, resulting from a different approach to compliance with international and national rules. While Finland is directing a lot of resources and powers into the fight against the use of tobacco products and takes its role in creating the conditions laid down in the Convention seriously, the efforts in Slovakia are debatable. Some progress and results have been achieved in the fields of labeling and tax collection, but the influence of the FCTC on the real behavior of smokers and the status of public health is difficult to prove. Slovakia should take a closer look at the Finnish implementation procedures, create specific priorities, and support the defined steps needed for their successful achievement by investing sufficient personal, material, and financial resources.

An overall and detailed survey at the national level is necessary to acquire current data and information on smoking habits of adult as well as young smokers. The current status of tobacco control policy is not at such a high level that Slovakia can afford to leave the majority of boxes in the WHO core questionnaire empty. More effort to see the situation realistically by the state representatives and people responsible for FCTC implementation is also crucial.

Further research should be done to identify the role of availability of cessation centers, medical treatment, and support from educated health professionals in decreasing smoking rates (art. 14, art. 26 of the FCTC) and also compliance at the level of groups within a state.

## 5. Conclusions

From the current point of view, it seems that Finland will be the first European smoking-free country before 2030. Slovakia has set out on this road and some successful steps can be seen, such as a good legislative basis, and it has reached partial milestones in the field of labeling and decreasing tax evasion. However, through the lens of public health, the fact that Slovakia is a Party to the FCTC does not in itself produce desirable results, especially looking at the continuing number of smokers, especially in the group of youth and minors, and in the area of establishing effective institutional capacities. The lack of current data and more detailed information still persists, so it is very difficult to identify the specific gaps and needs which could be addressed and covered with the assistance offered by WHO agencies. Raising cigarette taxes, implementing comprehensive smoke-free air laws (SFALs), banning all tobacco advertising, promotions, and sponsorships, and funding comprehensive tobacco control programs, particularly those including media campaigns, are highly effective strategies for reducing smoking prevalence. Cessation treatment policies and prominent graphic health warnings are likely to be especially effective in increasing quit success when combined with other policies that increase quit attempts [96].

There is a discussion among scientists about the concept of compliance, which can be seen from various angles and sometimes it is difficult to choose which interpretation should be used. This is probably another emerging question, whether it is not time to rethink why international law really matters and if “compliance” should not be replaced with the concept of “interpretation” [24]. To conclude, it is necessary to take into account that data and expert knowledge themselves are not enough for successful policy-making; much still has to be done to influence public opinion and enable coalitions to form with the same aim [97].

## Figures and Tables

**Table 1:** Slovakian and Finnish institutional frameworks for tobacco control policy (governmental and non-governmental).

Country/Type	Institution	Tasks/Activities	Established/Now
**Slovakia**			
Governmental	National Coordinator on Tobacco Control	N/A	1998/dissolved
	National Coordination Committee on Tobacco Control	N/A	2000/dissolved
	Tobacco Control Center	N/A	2004/dissolved 2006
	Public Health Authority of Slovakia	General drug prevention	2007/existing
	Regional Public Health Authorities (36)	General drug counseling	2003/existing
	Counseling Centers of Health (18)	Counseling	2009/N/A since 2009
	Slovak Regional WHO Office	Providing guidance, building up local relationships to implement technical cooperation, making standards and agreements	1994/existing
Non-governmental			
	National Coalition on Tobacco Control	N/A since 2007	2002/N/A
	Stop Smoking	N/A since 2012	2000/N/A
	League Against Cancer	Cancer prevention	1990/existing
	Slovak Medical Society	General drug prevention	1969/existing
	League of smokers and non-smokers	N/A	2003/N/A
**Finland**			
Governmental	National Institute for Health and Welfare	National tobacco and nicotine policy guidance	2009/existing
	Valvira (national agency)	Supervision of advertising ban and labeling	2015/existing
	Regional State Administrative Agencies (6)	Quality management (including healthcare)	2013/existing
	Ministry of Social Affairs and Health	Priorities, guidance and supervision	1917/existing
National Organization	ASH Finland	Tobacco policy expert	1989/existing
Non-governmental			
	Tobacco-free Finland 2030 network	Strategic anti-tobacco policy	2008/existing
	Filha	Clean air/fight against tuberculosis	1907/existing
	SOSTE	Well-being, health support	2012/existing
	Duodecim	Education of doctors	1881/existing
	EHYT	Substance abuse prevention	2011/existing
	Doctors against tobacco	Tobacco use prevention	1994/existing

N/A—not available; ASH—Action on Smoking and Health; Filha—Finnish Lung Health Association; SOSTE—Finnish Federation for Social Affairs and Health; EHYT—Finnish Association for Substance Abuse Prevention. Years indicate the date of establishment of the institution. Source: own processing based on data available on websites of the mentioned institutions (2019).

**Table 2:** Checks and monitoring actions in Slovakia.

Institution	Type of Action	Year
Ministry for Health	Review of non-smoking protection measures and compliance with the law;	2012–2014
	Tobacco, alcohol and drugs (TAD) survey at primary, secondary schools and universities;	2013, 2014
Labor Inspectorates with Regional Health Authorities	Checking compliance with the law on the protection of non-smokers and providing information on the possibilities of complaints;	2012, 2013, 2014
Ministry for Defense	Monitoring the prevalence of smoking in students of a selected university;	2012–2014
State Veterinary and Food Administration	Control of labeling of tobacco products;	In 2014
	Control of the production, storage and transport of tobacco products;	In 2014
	Control of the ban on the sale of tobacco-like products;	In 2014
	Control of ban on the sale of tobacco products; monitoring the prevalence of smoking in selected budget sector organizations	In 2014
Slovak Trade Inspection	Labeling, ban on sale of tobacco-products to minors	2013, 2014, 2018
Ministry for Transport and Construction	Monitoring the prevalence of smoking in selected govt-funded organizations in the sector	2012–2014

Source: own processing based on the Report on Fulfilment of the National Action Plan for Tobacco Control for the period 2012–2014 and Legal News website (2019).

**Table 3:** Implementation of the Framework Convention on Tobacco Control (FCTC) articles in Slovakia and in Finland.

FCTC Article	Slovakia	Finland
5.1 and 5.2	No responsible institution/missing answers	Well-functioning institutional structure/strategic documents adopted
5.4 and 5.5	Many answers N/A	Active in the field of cooperation at regional and international level
12	Call for raising awareness	Media planning, assessment of implementation and effectiveness
14.2	A lack of designated funds for tobacco control	A lack of designated funds for tobacco control
16	Decreasing tendency in the selling tobacco products to minors	Decreasing tendency in the selling tobacco products to minors
20, 21, and 22	Low level of cooperation and exchange of information/N/A	High level of declared cooperation and exchange of information

Source: own processing based on the 2018 Core questionnaire of the reporting instrument of FCTC (2020).
